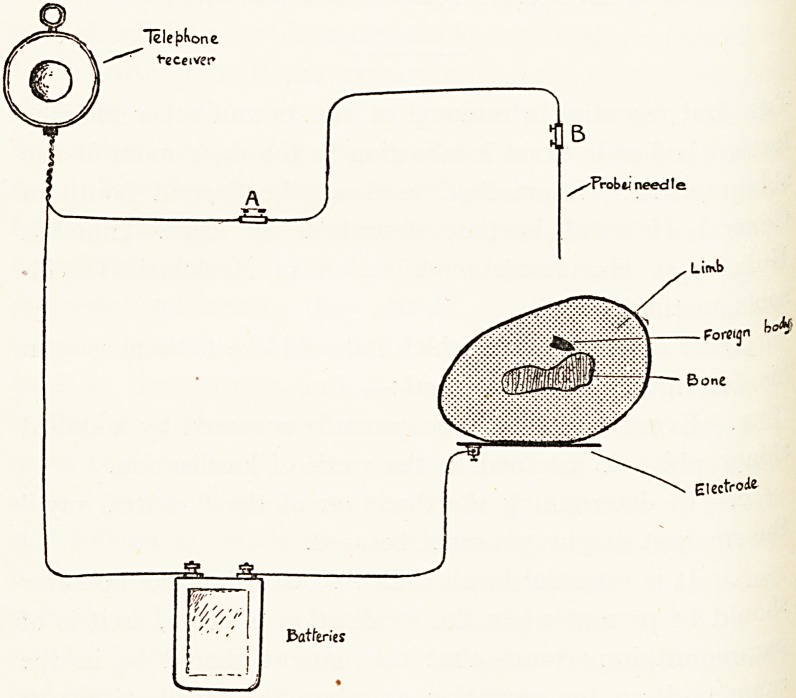# Removal of Bullets and Other Metallic Foreign Bodies

**Published:** 1915-12

**Authors:** R. G. Poole Lansdown

**Affiliations:** Major, R.A.M.C. (T.F.), 2nd Southern General Hospital


### REMOVAL OF BULLETS AND OTHER METALLIC FOREIGN BODIES.

BY

R. G. POOLE LANSDOWN, M.D., B.S. Durham,

*Major, R.A.M.C. (T.F.), 2nd Southern General Hospital.*

---

THE first essential in removal of bullets and other metallic foreign bodies is exact localisation in the dark room of the skiagraphist. The method used at the Second Southern General Hospital is that described by Capt. Thurstan Holland as Hampson's modification of Mackenzie Davidson's method.[1]

There are four points which I should like to emphasise in his description of this method :—

1. Every case should be carefully screened by a skilled skiagraphist accustomed to the work of localisation.

2. In determining the direction of the "central ray" the smallest diaphragm must be used.

3. It is essential in all difficult cases that the operator should be present when the localisation is made, as it is of paramount importance that the patient should be in the same position for operation as when the localisation was carried out.

4. The localisation should be made as short a time as possible before operation, owing to the liability of the metallic body to shift its position.

Various methods of marking the skin have been tried and found wanting. The marks should be small and not capable of being washed out or got rid of in preparing the skin for

[1] *Archives of the Röntgen Ray*, No. 175, Feb. 1915, pp. 310-16.

operation. The method of marking found most satisfactory is a small cross made with a sterilised surgical needle.

When the patient is anæsthetised and ready for operation the second essential in successful removal is to be able exactly to identify the position of the bullet before incising the skin. At the Second Southern General Hospital we use an apparatus figured in the accompanying diagram. The diagram sufficiently explains its use. In actual practice the electrode, shown attached to the limb being explored, is always attached to another limb. The probe-needle and portion of wire beyond the junction A are sterilised. The telephone receiver is held against the telephone operator's ear, the current turned on and the probe-needle passed through the tissues in the direction indicated by the marks previously made on the skin by the skiagraphist. When the needle touches the metallic body a characteristic "tap, tap, tap," is heard in the receiver.

The advantages I claim for this particular type of bullet searcher over any other pattern are :—

(i.) There is only one pole to strike the metal.

(ii.) The pole consists of a sharp, long needle.

(iii.) No skin incision is necessary for the detection of the metallic foreign body.

(iv.) No damage is done to the tissues, as if no metallic foreign body is detected no incision is made.

(v.) That portion of the apparatus which approaches the area of operation is simple and inexpensive, easily replaced at once, and can be readily sterilised.

As soon as the operator in charge of the telephone notifies that the needle has struck metal the current is turned off and the skin incision is made, with the needle as the centre point of the incision. The length need not be more than comfortably to admit an exploring finger if necessary to insert it. The centre of each side of the incision is then fixed to the edges of the towel by towel-forceps, which serve also as retractors. The incision is then carried inwards, using the needle as a guide until the metal body is struck by the scalpel. The bullet or piece of shrapnel is then grasped by a pair of forceps or is caught between the tip of the little finger and a gall-bladder scoop and lifted out.

The only retraction of tissues permissible in addition to that obtained by the towel-clips is carried out by the assistant with a small, flat retractor on the side of the incision away from the operator. Any undue use of retractors or blunt dissectors or free use of a finger, more often than not, results in the bullet being drawn or pushed to one side of the line marked by the telephone needle. Should this happen, the current must be switched on again and attempts at extraction deferred until the telephone operator signifies that the needle is in contact with the bullet. Care is needed to protect the needle and wire from contact with any surgical instruments in the field of operation, as such contact will at once convey a message of metallic finding to the telephone operator. Where the bullet is situated in an abscess cavity pus escapes as soon as the scalpel enters the abscess. If the abscess is a large one the position of the bullet may be considerably altered by the collapse of the cavity. An index finger will then easily detect the bullet as it is lying free in the cavity uncovered by muscle.

When the bullet is embedded in a large muscle it is extremely easy to miss it altogether for two reasons :—(i.) it is practically impossible, when it is covered by even a thin layer of muscle tissue, to feel the bullet unless the operator can fix it between his exploring finger and a firm resisting body such as a bone. (ii.) when the needle is used in searching for the bullet with the current turned on, it causes contraction of the muscle in which the bullet is embedded. The contraction will often carry the bullet to one or other side of the guiding line laid down by the skiagraphist, and unless the depth of the metal has been carefully marked by the surgeon on his needle before commencing, its point will pass too deeply and the bullet be missed.

The first of these possibilities of failure is quite easily overcome or avoided by following the rule to use retractors and exploring fingers as little as possible, or not at all until the knife following in the line of the needle itself strikes the bullet.

The second possibility is overcome as soon as the cause of the failure to strike metal is realised, the needle being partly withdrawn and then pushed down to the measured depth to one or the other side of the skiagraphic line. Sometimes if the bullet is in muscle it may become displaced between the time of localisation and operation.

A third cause of failure is peculiar, and takes a little time to tumble to. The foreign body stated by the wounded man to have resulted from a gunshot wound, and which is well shown in skiagram and felt by the operator with his exploring telephone needle, fails absolutely to give the signal of metallic finding to the operator. The possibility of the foreign body being a piece of stone or other material somewhat opaque to X-rays must be borne in mind. This was brought forcibly to my notice by the suggestion of Capt. Taylor in a case in which a piece of material in the chest-wall on three different days failed to give a metallic finding signal to the telephone operator.

There is nothing more depressing for a surgeon than to know that he has failed in accomplishing the object with which he commenced his operation, but where the operation has not actually been commenced the depression need not be present, for no harm has been done to the patient. Unless I can obtain the metallic finding signal after a careful search with the needle between the fixed points laid down for me by the skiagraphist, I send the patient back to bed and have a fresh localisation carried out next day.

We have often to decide whether or not a bullet or piece of shrapnel is best removed or best left alone.

1. All superficial metallic foreign bodies which cause any discomfort or are likely to give trouble from their presence should be removed.

2. In gun-shot wounds which do not heal, or where sepsis continues, if a foreign body is present it should be removed.

3. Deeply-situated bullets or pieces of shrapnel should be removed from limbs if of large size and causing any inconvenience. If causing no inconvenience, leave alone.

4. Those situated within the thorax, unless easily reached, are better left alone. I have seen two cases, with no symptoms, in one of which a round shrapnel bullet was lying on the summit of the arch of the aorta, in the other a piece of shrapnel casing was lodged on the right side of the arch. Both were left alone.

5. In the abdomen, if found on repeated localisations to be in the same position, the bullet should be removed. If on repeated examinations it is found in different parts of the abdomen, it is probably free in the peritoneal cavity, and can be removed generally from the pelvis if the man has been walking about. Bullets so placed are treated much in the same way as other foreign bodies in the abdomen. Dame Nature wraps them round with adhering coils, and eventually they will be extruded *per vias naturales*.

6. In the head. Those situated in the scalp, in the face, or in the bony case of the skull should be removed, and in the latter portions of depressed inner table looked for. If situated within the brain itself it is impossible to lay down general rules. If the wound is septic around the bullet, a large area of brain necrosis may be extruded, and the patient dies suddenly. Where situated immediately under the skull, provided it has been carefully and accurately localised, the bullet should be removed by the shortest route.

To sum up :—

1. Never attempt to look for a bullet or shrapnel without having had it carefully localised, and if likely to be a difficult case be there yourself.

2. Make no incision unless the metal has been located by the telephone needle, but send patient back to bed for fresh localisation.

3. Disturb the deep parts of the wound as little as possible with retractors, fingers or blunt dissectors until the foreign body is exposed, and then lift it out with care, not to damage the tissues through which it is withdrawn.

## Figures and Tables

**Figure f1:**